# Optimizing intervention dissemination at scale: A qualitative study of multi-sector partner organization experiences

**DOI:** 10.1093/tbm/ibae042

**Published:** 2024-08-31

**Authors:** Samuel Cassar, Jo Salmon, Anna Timperio, Byron J Powell, Jacqueline Della Gatta, Jiani Ma, Harriet Koorts

**Affiliations:** Deakin University, Geelong, Institute for Physical Activity and Nutrition, School of Exercise and Nutrition Sciences, Burwood, VIC, Australia; Deakin University, Geelong, Institute for Physical Activity and Nutrition, School of Exercise and Nutrition Sciences, Burwood, VIC, Australia; Deakin University, Geelong, Institute for Physical Activity and Nutrition, School of Exercise and Nutrition Sciences, Burwood, VIC, Australia; Center for Mental Health Services Research, Brown School, Washington University in St. Louis, St. Louis, MO, USA; Division of Infectious Diseases, John T. Milliken Department of Medicine, School of Medicine, Washington University in St. Louis, St. Louis, MO, USA; Center for Dissemination and Implementation, Institute for Public Health, Washington University in St. Louis, St. Louis, MO, USA; Deakin University, Geelong, Institute for Physical Activity and Nutrition, School of Exercise and Nutrition Sciences, Burwood, VIC, Australia; Deakin University, Geelong, Institute for Physical Activity and Nutrition, School of Exercise and Nutrition Sciences, Burwood, VIC, Australia; Deakin University, Geelong, Institute for Physical Activity and Nutrition, School of Exercise and Nutrition Sciences, Burwood, VIC, Australia

**Keywords:** schools, dissemination, research–practice partnership, translation, scale up

## Abstract

For population-wide impact of interventions, evidence-based programs must be successfully disseminated and adopted at scale. Research–practice partnerships can legitimize programs and support organizational adoption, however, ways of leveraging the contributions of partners during dissemination processes are less clear. *TransformUs* is a school-based physical activity and sedentary behaviour program, and since 2018, has been disseminated at scale to all primary schools in Victoria, Australia, in partnership with 16 state and national organizations. The study objective was to investigate the experiences of partner organizations disseminating *TransformUs* within their networks, and factors associated with awareness and adoption of the program in schools, from the perspective of partner organizations. Semi-structured interviews with representatives from 15 (out of 16) partner organizations in 2019 involved in disseminating *TransformUs* state-wide. An interview guide was informed by the RE-AIM framework. Interviews were audio-recorded, transcribed, and coded anonymously. Data were analysed thematically. Four themes (and 13 sub-themes) were identified: (i) organizational barriers and facilitators to dissemination; (ii) implementation support for partners; (iii) overall research experience; and (iv) dissemination strategy. Partners used multiple dissemination channels to increase adoption, and strong alignment between *TransformUs* and the partner organization’s goals appeared to enable dissemination. Partners outlined several local, regional, and state organizations to partner with, and offered suggestions regarding preferred content and timing of dissemination activities for their networks. Researchers planning research–practice partnerships should proactively consider organizational barriers partners may face during dissemination. Regular communication and feedback on dissemination outcomes may contribute to positive research–practice experiences and allow for tailored dissemination strategies.

## Teaser

Ways of disseminating an efficacious school-based program targeting improvements in children’s physical activity and sitting time at scale.

**Implications**

**Practice:** Organizations and relevant parties should consider their reach among other organizations and ability to promote, and cross-promote interventions within their relevant systems.

**Policy:** Policymakers should consider the key individuals and organizations they wish to partner with for disseminating interventions, as well as how to align partnerships to overarching policy targets within the system.

**Research:** Researchers should plan actions to enhance the efficacy of partner-driven dissemination on intervention uptake and implementation through deliberate planning and action.

## Introduction

Despite the numerous health [1], financial [2], and environmental [3] benefits of physical activity in childhood, the majority of children worldwide are not meeting physical activity recommendations [4]. Whilst evidence-based interventions to improve physical activity are available [5, 6], they are often not translated into everyday practice and certainly not at a scale capable of yielding a population-wide impact [7]. There are many factors underpinning inadequate research–practice translation, including ineffective dissemination of interventions into practice [8]. Consequently, the dissemination of physical activity recommendations and programs have been advocated [9–11].

Dissemination can be defined as a deliberate communication strategy targeting intervention end-users [12] as opposed to a passive strategy that relies on individuals to adopt and implement new interventions [12]. Dissemination research attempts to identify effective approaches that improve the uptake of evidence [13]. However, physical activity researchers often lack the expertise, time, project funding, and organizational support required to engage with relevant parties [14]. In a recent editorial, it was argued that effective research–practice partnerships are essential in dissemination efforts to increase population levels of physical activity [15]. Research–practice partnerships which can be defined as ‘long-term collaborations between practitioners and researchers that are organized to investigate problems of practice and solutions for improving schools and school districts’ [16]. In school-based programs in particular, partnerships with and between health, education, and other associated sectors are necessary to support dissemination activities at scale [17].

Within schools, the research-to-practice gap often relates to failed implementation of school-based programs [18], perhaps due to a myriad of implementation barriers [19]. Guides on disseminating interventions often describe the importance of engaging relevant parties and purport the benefits of working collaboratively in the early phases to increase buy-in during dissemination, although there is a lack of step-by-step guidance on how to do so [20]. The PRACTIS guide has made steps toward addressing this gap, highlighting how participatory and co-production approaches may be utilized to increase the uptake of physical activity interventions [21]. Consideration of diverse relevant parties becomes more pivotal for interventions spanning multiple systems where potential dissemination activities may differ from partner to partner (e.g. raising awareness/education of end-users, sharing with their networks). The mapping and tailoring of promotional activities via partner organizations thus provide valuable information on the frequency, reach, content, audience, mode, and space allocated to dedicated dissemination activities. Whilst multisectoral partnerships may be essential in dissemination efforts [15], little is known regarding how relevant parties support the dissemination of interventions, or their potential role in increasing the likelihood of subsequent end-user adoption [22].

This paper presents findings from a qualitative evaluation of the state-wide dissemination of an efficacious primary school-based physical activity and sedentary behaviour intervention (*TransformUs*) across Victoria, Australia [23]. *TransformUs* is the first school-based physical activity intervention of its kind to be implemented at scale and offered to all primary schools in Victoria, Australia [24]. This study aimed to explore the partner organizations’: (i) experiences of disseminating *TransformUs* to their professional networks; (ii) perceptions of *TransformUs* dissemination activities; and (iii) views on improving program dissemination.

## Methods

### Overview of TransformUs

The objective of *TransformUs* is to promote children’s physical activity and break up prolonged sitting using pedagogical and environmental strategies within the classroom, school, and home setting [23]. Since 2018, *TransformUs* has been available to all Victorian Primary Schools (~1900) via an online platform as part of a hybrid implementation-effectiveness trial [24]. State-wide implementation of *TransformUs* involves dissemination of the intervention via research–practice partnerships. The 14 implementation and scale-up strategies underpinning the *TransformUs* state-wide roll out have been published previously [24]. The scale-up approach covers five theory-based strategies that relate to the dissemination of the program, including (i) formative work with relevant parties; (ii) creation of coalitions and networks for program/policy advocacy; (iii) multiple dissemination routes/channels; (iv) alignment with existing state-level initiatives and guidelines; and (v) monitoring and evaluation to adjust the scaling strategy and provide feedback to support schools.

In the present study, the dissemination of *TransformUs* involved research–practice partnerships, partners included 16 key organizations who actively promoted the program within their professional networks, and provided input on trial and evaluation design. These organizations represent education, health, and sport and recreation government departments; professional teaching associations; peak public health bodies; not-for-profit health organizations; non-government education organizations; a state statutory authority; and a professional sporting club. Five of the 14 implementation and scale-up strategies relate to dissemination efforts and include formative work with relevant parties (e.g. workshops, co-development of program materials), the creation of coalitions and networks for program advocacy (e.g. engaging state-level decision-makers and opinion leaders to form partnerships), utilizing multiple dissemination routes and channels (e.g. media releases, newsletters, conferences, etc.), aligning with existing state-level initiatives and guidelines (e.g. the Victorian Achievement Program [25] and Education State Targets [26]), and ongoing monitoring and evaluation to adjust the scaling strategy (e.g. regular informal communications with program partners, regular more formal group meetings, and formal interviews with partners regarding dissemination activities; see a full list of strategies in Appendix 1).

### Design

A qualitative study involving semi-structured telephone interviews with key contacts representing *TransformUs* partner organizations was conducted (*n* = 15). Interviews were conducted in line with recommendations set out for qualitative methods in implementation science, which allow for in-depth responses relating to the inherently complex processes involved in implementation, such as dissemination [27]. An overview of the study in accordance with the Standards for Reporting Qualitative Research (SRQR) is presented in Appendix 3.

### Participants

Purposeful sampling [28] of key contacts within all *TransformUs* partner organizations (*n* = 16) was conducted. Individuals who held specific responsibilities associated with disseminating and promoting the program over the last 2 years were invited to participate. Potential participants were contacted via email and sent a Plain Language Statement and consent form. Compensation for participation was not offered. All participants provided verbal and signed consent. Recruitment ran from 4 September 2019 to 1 October 2019.

### Theoretical framework and interview guide

An interview guide (see Appendix 2) was developed to explore experiences of disseminating *TransformUs* and partnering with project investigators in the research team. This semi-structured interview guide was informed by the RE-AIM framework [29], a well-established implementation outcomes framework used to evaluate public health interventions [30]. Key RE-AIM dimensions covered in the interview guide included: Reach (characteristics of organizations that promoted the dissemination of *TransformUs*), Effectiveness (perceived success of dissemination strategies), Adoption (factors relating to the adoption of *TransformUs* in schools), Implementation (organization’s involvement in supporting schools to deliver *TransformUs*), and Maintenance (organization’s willingness to continue promoting *TransformUs*).

The interview guide included 24 questions and a list of prompts for each question to ensure consistency between interviews. In addition to interview questions covering the five domains of the RE-AIM framework, participants were asked generic descriptive questions about their experience and role within the organization and involvement in disseminating *TransformUs*. Recommendations for improving the program and dissemination support for partners moving forward were also sought. Interviews were conducted via telephone by a trained qualitative researcher (S.C.) and lasted between 25 and 50 minutes (Mean = 38 minutes). All interviews were voice-recorded and transcribed verbatim by an external online transcription company.

### Analysis and interpretation

Analyses were undertaken using a framework analytic approach [31]. This approach allows for consideration of both *a priori* codes (according to the RE-AIM framework) and emergent codes (e.g. relating to the research partnership involvement). Two coders (S.C. and M.C.—see acknowledgements) independently analysed the interview transcripts according to a coding manual developed by S.C., J.S., A.T., and H.K. The RE-AIM concepts were used as starting points for code generation, from which themes and sub-themes were generated via discussion and consensus decision (S.C. and M.C.). Data familiarization was conducted on interview transcripts with each independent coder first listening to the interview recording and reading interview transcripts twice before qualitative data coding was completed in the NVivo software to find key themes and quotes. In addition, both coders kept a research journal throughout the analysis to aid reflexivity (i.e. the research process and the researchers position within this context) [32]. Coding discrepancies were resolved by discussion and consensus decision (S.C. and M.C.). To promote the trustworthiness of the coding and analysis process, an independent researcher (M.C.) who had no prior relationship with the partner organizations provided an additional layer of objectivity and helped to validate the findings. Regular debriefing sessions were conducted with the independent researcher and the primary research team to reflect on the coding process, discuss potential biases, and refine the analytical framework.

## Results

Interviews were conducted with one key contact from each of the 15 (out of 16) *TransformUs* partner organizations. One partner organization declined participation as they felt it was beyond the scope of their involvement in the program. Participant demographic characteristics are presented in Table 1 and highlight the comprehensive organizational coverage of relevant parties within the Victorian educational, health, and physical activity systems. Participants represented a variety of roles, including with government policy, as heads of professional teaching societies, educational leadership in curriculum development and school leadership, non-profit health promotion, a professional sporting club, and community health. For many of the partners, engagement in the program commenced well prior to the 2018 launch. The results described below highlight where the scale-up strategies occurred and provide suggestions for improvements to the dissemination of the program. A summary of the dissemination strategies is also provided in Appendix 1.

**Table 1:** Participant characteristics

No of participants	Organisation type	Partners joined *TransformUs*	Time in role
4	Peak public health bodies/not-for-profit health organizations	Nov 2015, Jan 2017, Aug 2017, Jun 2019	Unknown
3	State government departments (Education; Health; Sport and Recreation)	Nov 2015, Jan 2017, 2018	Ranging from 1.5 years to 20 years
3	Professional teaching associations	Nov 2015	Unknown
2	Non-government Education organizations	Nov 2015, Jan 2017	Ranging from 2-2.5 years
1	State statutory authority	Jan 2017	Unknown
1	Sporting club	Jun 2018	Ranging from 2 years 9 months to 13 years
1	Federal government department (Sport)	Jun 2018	Unknown

### Dissemination activities

Partner organizations reported that they had disseminated the program to school leaders, classroom teachers, physical education and health teachers, and school health/well-being teams, ensuring multiple touch points within schools (Strategy 3, Appendix 1). Dissemination activities described throughout interviews included program information in the following forms: E-blasts to partner organization members, schools, and teachers, social media engagement (e.g. tweets and retweets advertising the program), running workshops, giving presentations, and providing trade stands at educational conferences. ‘Tip of the week’ style promotions for teachers were provided by the teacher professional societies. Newsletters were also provided to health and physical education teachers, and informal discussions were held with school leaders, teachers, and regional administrators.

**Figure 1:**
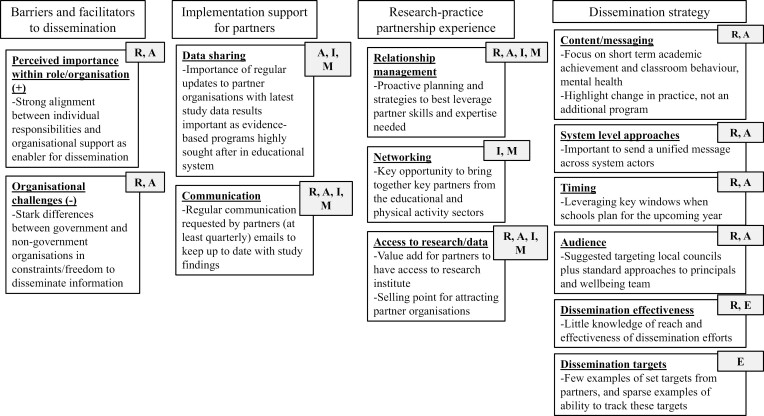
Theme and sub-theme descriptions, linked to RE-AIM domains

Four themes and 13 sub-themes emerged from the interview data (Fig. 1), which related to (i) organizational barriers and facilitators to dissemination; (ii) requested implementation support for partners; (iii) research–practice partnership experience; and (iv) the dissemination strategy.

### Theme 1: Organizational barriers and facilitators to dissemination

#### Perceived importance within role/organization

By aligning the program with state-level initiatives and policies (Strategy 10, Appendix 1), it was hypothesized that the program would be more likely to gain support from key actors in the educational sector. Most participants perceived that *TransformUs* was an important program that helps their organization meet its goals/aims: *‘So, we really, really are very, very closely aligned. We are on the same page in terms of what we’re trying to achieve and that’s I suppose why we’ve partnered and why we’re more than happy to promote TransformUs’. (Professional teaching association).* In this instance, the perceived importance facilitated the adoption of the program.

One organization highlighted that whilst the program was not integral to for them to achieve their organizational mission, they endorsed implementation in schools: *‘So TransformUs is an interesting project in its own right but its neither here nor there, we’re ambivalent or down the middle, internally with us. I think it’s a worthy project and I hope it does tick along in schools’ (Non-government Education Organisation).*

#### Organizational challenges

An important aspect of the dissemination strategy (Strategy 14, Appendix 1) was to regularly monitor progress and provide support where needed. In response to describing any barriers faced in disseminating *TransformUs* among their professional networks, there appeared to be a clear divide between government and non-government organizations. Non-government partners did not describe organizational challenges to disseminate the program on the research team’s behalf, with one participant stating: ‘*No, no, we’ve promoted it through all of our different means. The only thing and like it’s not really a challenge, is whether or not there would be a place for us to be more proactive’ (Professional teaching association).*

Representatives of government department partnerships found themselves under different constraints and having to play a juggling act to demonstrate fairness between the numerous programs they promote and disseminate. Additionally, there were restrictions within government departments to minimize the information burden on schools that was described as a barrier to dissemination: *‘We’re quite restricted in direct communication to schools at the moment. There’s a big push to minimize the pressure on principals. So, we’re in a phase where the Department is trying to streamline communication, sort of triage what is most urgent’. (State government department).*

### Theme 2: Requested implementation support for partners

#### Data sharing

When prompted about the support partners believed would help them to disseminate *TransformUs,* there was a desire for data sharing, with regular and more periodic study updates considered to be helpful in enhancing the adoption and implementation of the program. Data sharing was described as providing content to enhance dissemination efforts, as actors in the educational system welcome information on evidence-based programs as leverage to gain early support (i.e. adoption), again underlining the importance of sharing data early: *‘I think once some of the data starts coming out, I think that that will be important to sort of share with the stakeholders because that’ll sort of give us, obviously, some leverage as well around being able to talk about potentially some of the preliminary results that could help with getting more support for the program’. (State government department).*

In addition, partners suggested it would be helpful if the *TransformUs* team could generate case studies of schools that are currently implementing the program (Strategy 14, Appendix 1). These could be disseminated and would give schools considering adopting the program an idea of how it looks in practice: *‘So, any case studies with photos, approvals… we could use to promote through our e-news, which goes to all schools. And they feature the good work schools are doing, and they often use that in other means, in other communications and reports’ (Non-government Education organization).*

#### Communication

In addition to data on results, participants described regular communication as another aspect that could be improved to enhance dissemination efforts. As part of Strategy 2 (Appendix 1), bi-annual meetings were planned, and informal communication (such as updates on project progress) was conducted regularly throughout the dissemination process. Regular communication was mentioned by the partners as a prompt and incentive to disseminate the program, with some participants noting a drop in the frequency of project updates in the previous 6 months (which occurred due to extended staff leave and delays in filling the position). *‘It’s really gone quiet. If you’re wanting us to do something, because I’ve got lots of things on and I’m not going to drive this...like, I think it needs to be driven by you guys and we really need to know what your dissemination plan is’ (Peak public health body).*

There were requests for monthly or quarterly updates in the form of emails and e-blasts which should include new developments, current recruitment data, and preliminary study findings to help maintain engagement between partners (i.e. maintenance).

### Theme 3: Research–practice partnership experience

#### Relationship management

Following suggestions regarding regular partner communication, some participants described the sense that research–practice partnership arrangements need to be led by the research team. This is an example of where the scale-up strategy (Strategy 1, Appendix 1) could have been clearer in setting expectations and roles for partner organizations. For example, improvements in managing expectations was identified by one partner who felt that the research team did not leverage their expertise in certain areas of the project: *‘So, yes, I just feel as though we have expertise that may or may not have been used as well as it could have’ (Professional teaching association).*

#### Networking

Many participants described the partnership as a valuable opportunity for networking between the education and physical activity sectors and the chance to get key organizations and people in the same room (Strategy 2, Appendix 1). For example, regular in-person project updates between the research team and partner organizations provided opportunities for informal conversations and broadened participants’ networks: ‘*It is a good networking opportunity in terms of keeping the physical activity and the education sectors interlinked’ (State government department).*

*That’s been a bonus in terms of broadening the networks available, for me, branching out to many other organisations. That’s definitely a bonus for our organisation. (Professional teaching association).*

#### Access to research/data

Partner organizations appreciated the opportunity to work closely with well-established research institutes and described this as one motivation for their involvement in the project. This highlights the importance of Strategy 2 (Appendix 1) in engaging partners in the research process through consultation, which contributes to organization’s willingness to continue promoting the program (i.e. maintenance). Participants also noted the research–practice partnership had added benefits in relation to accessing other novel research data connected with the research institute: ‘*The value we’re getting out of this partnership is not just a chance to see new programs on the ground, and connect them to schools to add greater value, but it’s also the associated research that you guys have around those programs. That’s contributing to a body of knowledge that’s really important for us, that we could never afford to fund as an agency’. (Not-for-profit health promotion organization).*

### Theme 4: Dissemination strategy

#### Content/messaging

Participants made several recommendations regarding the messaging and content of dissemination activities (Strategy 3, Appendix 1), including to showcase the benefits of physical activity to learning, concentration, and overall energy of school children throughout the day: ‘*That is the obvious sort of point around the benefits to their learning from physical activity and their level of concentration and energy etcetera and readiness to learn in the day’ (State government department).*

Others spoke of the need to reframe messaging that provide meanings that schools may value:

*It’s just a matter of how we can look at it from a real strength-based approach to, okay, if kids are active, we will have these outcomes in their physical, in their mental health, and in their academic performance. (Professional teaching association).*

Interviewees further described the need to use case studies to show that *TransformUs* is not time or resource-intensive and makes teachers’ lives easier. One participant supported the need to make sure to promote *TransformUs* as a change in practice, rather than an additional program: *‘I guess when you brand something in a certain way, and give it a name, and give it its own sort of space, which sort of TransformUs has, maybe it’s perceived as a program as such, when it’s not intended to be another program, it’s actually just a different way of doing things’ (State government department).*

There was also a suggestion to leverage existing state policy documents already in place:

*You’ve got the education sector target around the physical needs of children, and that’s for you to try and promote that. (Professional teaching association).*

#### System level approaches

Related to the idea of leveraging State policies (Strategy 10, Appendix 1), was the argument from several participants that it is important to create a systems-level approach to messaging which includes a unified message from the relevant government departments: ‘*I think the power comes from having a joined-up message across Education, Health and Sport Ministers and an anchor point’ (State government department).*

Furthermore, it was recommended by participants that ensuring a broad range of partners all send a unified message to the public (e.g. advocating for the benefits of physical activity to learning), as it sharpens the focus to a smaller number of high-impact programs and may lead to more dissemination success across the system: ‘*I think that’s the only way that any of us can get any traction in the future, is to probably sing with one voice, and sing it loud. And ensure that we’re not trying to necessarily compete with each other, but how we each add value into the system together, to achieve the outcomes we’re probably all seeking for, which are quite similar.’ (Not-for-profit health promotion organization).*

#### Timing

The timing of messaging to schools about adopting *TransformUs* was deemed crucially important to influence adoption decisions. The end of term four in the Australian school year (October, November, and December) when planning for the next school year begins, was consistently mentioned by participants. For example: *‘Around that October, probably early November, they’re [Schools and school leaders] starting to gear up for next year. They’re looking at staffing, who’s leading what other projects, all that. The timing is really important. I think probably for our point of view that maybe early to mid-November is probably a good time’ (Non-government Education organization).*

#### Audience

Participants provided mixed responses in relation to the best possible audience for dissemination efforts, perhaps due to their broad ranging organizational foci. One suggestion related to targeting dissemination efforts to local councils and officers who have direct access to teachers and schools, thereby enhancing the reach of the program: *‘So you’ve got embedded officers in the councils that understand the need for physical activity and understand how to support the schools in their area to do it’. (Peak public health body).*

Others proposed contacting principals directly due to their decision-making power, e.g.:

*I’ve always found in the past is that talking to principals can really help as opposed to talking to a PE teacher or a classroom teacher or something like that (Sporting club).*

#### Dissemination effectiveness

Participants found it difficult to provide specific feedback on the effectiveness of their dissemination efforts. Few partners knew whether their dissemination activities were being successful or having an effect on promoting schools’ adoption of the program, e.g.: *‘It’s hard to know the impacted scale. Certainly we’ve had schools, they’re probably one of those that are quite progressive and have really strong leadership’ (State government department).*

In addition, partners thought they were reaching schools with their messages, but were not sure if it was enacting change: *‘I have no visibility of how successful comms [communications] have been in getting the message out there. But I imagine it’s getting to principals, but perhaps, the message is not getting out to teachers’ (State government department).*

#### Dissemination targets

The majority of partner organizations spoke of not having any set internal goals relating to their dissemination activities, or to the potential uptake and adoption of the program, with most statements reflecting the following statement: *‘How many of them are going to want to, or take action to work on, physical activity and movement in the next 12 months? It’s really a…would be a guessing game’. (Peak public health body).*

## Discussion

This study explored the process of ongoing dissemination of an evidence-based intervention being offered at scale, from the perspective of key individuals from partner organizations. Findings highlight alignment between the dissemination process experienced by partner organizations and the scale up and implementation strategies. The discussion below outlines key lessons and ways these may be operationalized in disseminating *TransformUs* and other similar evidence-based interventions.

1) *Organizational barriers and facilitators to dissemination*

One enabler of dissemination was the strong alignment between the intervention goals and those of the partner organizations. When considering potential organizations with which to partner in an implementation or scale-up study, it is paramount to clearly outline where and how the proposed intervention aligns with the core principles of each organization [33]. This may encourage partners to take ownership of their role in the intervention’s success (i.e. the dissemination of the program within their professional networks). In one instance, a partner organization mentioned that *TransformUs* was not a core goal of their organization, but this did not hinder their involvement in the program thus potentially highlighting there may be multiple reasons for involvement in the program (e.g. other recognized benefits included the chance to form networks with other key organizations in the sector or alignment with macro-level policy decisions). In the case of *TransformUs*, physical activity targets set by the state education department [34], and a joint ministerial statement (between education, sport and recreation, and health) on the importance of physical activity for children and young people in which the State Government recommended *TransformUs* to schools may have provided an impetus for partners to become involved. These developments may also signpost to organizations in the above sectors that physical activity promotion in schools will be an outcome of interest moving forward [35]. Despite alignment with organizational priorities, it is important to note that organizational challenges (such as having to liaise with a communications team to approve and schedule dissemination efforts) existed for some partners, particularly from the government sector who were constrained in frequency and content of dissemination given the range of programs they promote.

Relationship management with partner organizations, particularly with regard to projects which involve relevant parties from various levels of the delivery system, is another critical aspect to consider [36]. The *TransformUs* intervention is delivered within the education system, with engagement of relevant parties at different levels of the education, health, and physical activity systems. The reality of engaging multiple partners in this manner is that each is likely to have their own competing priorities and expectations for the project [8]. Developing and maintaining strong partnerships and keeping regular and consistent communication is integral for partners to sustain dissemination activities. The suggestions from partners highlighted a need to alter the original plan to hold bi-annual partner meetings to more frequent quarterly project updates to ensure communication channels remain open and regular.

2) *Implementation support for partners*

Participants in the study requested regular sharing of study data as a support for their dissemination work. This is noted in the wider literature regarding the uptake of evidence in schools, whereby practitioners (partner organizations in this instance) should be invited to collaborate on producing evidence resources [36]. Requests from partners for more frequent data sharing were explained as a way for the project team to maintain engagement with partners and described as a promising way to drive uptake of the program in schools.

3) *Research-practice partnership experience*

Participants in the study described their experience of the research-practice partnership as a positive one that helped their organizations. Such partnerships are key to sustained dissemination efforts, as the responsibility for most aspects of dissemination are argued to be undefined or underfunded in public health research [37]. Participants also valued the opportunity for a range of *TransformUs* partner organizations to work collaboratively on a shared goal, which facilitated new networks between relevant parties that can lead to other outcomes or assist their organization in other ways. Therefore, promoting networking opportunities are a potential recruitment strategy that could be used as an incentive by implementation and scale-up initiatives when seeking partners.

4) *Dissemination strategy*

The granular aspects of how partners can help with dissemination (e.g. who does what when, specific times/events where some but not all partners help out) is important to be laid out in a dissemination plan [8]. The purpose of Strategy 3 was to facilitate dissemination via multiple channels, and suggestions on the specific aspects of dissemination allowed for the identification of key audiences and the best timing of dissemination efforts. Partner organizations requested the provision of content for dissemination activities and recommended the program team to consider that if organizations from multiple sectors and interests promote the intervention using consistent messaging, a more diverse range of schools who engage with these organizations are likely to be reached. It has been argued that audience characteristics are important in shaping a dissemination strategy [8]. In the current study, partners discussed the importance of targeted dissemination that considers which channels are most important for each audience.

Consistent with evidence that each audience will be affected differently depending on the message [38], a key finding from partners was the suggestion that school audiences are more inclined to act based on case studies than other more traditional forms of dissemination (i.e. newsletters). In particular, key messages that target the end-user knowledge needs [36], and centre on the benefits of additional movement embedded in the school day can bring to students’ mental health and well-being, and the impact this has on overall academic achievement were deemed of great interest to schools. Indeed targeted dissemination strategies which consider the medium (i.e. case studies), content of key messages for specific audiences (i.e. program effects on classroom behaviour for teachers), and timing that is determined by the target audience and their organizational structures are central to successful dissemination [39].

The intent of Strategy 14 was to be able to make iterative improvements to the program and to the dissemination plan as the project progresses [24]. Understanding the organizational barriers and facilitators raised by the partner organizations and the feedback they provided on the dissemination strategy described here, was used to adapt the dissemination plans. This process is ongoing and reflects the importance of tailoring dissemination strategies to the determinants on the dissemination process and tailoring, with input from relevant parties [40].

## Strengths and Limitations

A strength of this study was the novel inclusion of program partners as participants to qualitatively gain a greater understanding of dissemination [41]. The use of a framework analytic approach [31] offered a systematic and flexible way to analysing qualitative data, which means that we were able to consider both *a priori* and emergent codes to create a new structure for the data that is helpful to summarize/reduce the data in a way that can support exploring partners’ experiences and perceptions of disseminating *TransformUs*. It is clear that public health researchers want their work to have an impact beyond traditional research outputs [42], and this study provides an insight into the ways to engage and manage research-partnerships to optimize the dissemination of interventions. A limitation was that several participants had only had exposure to *TransformUs* for a short time within their organization and may not have been able to reflect on their organizations entire experience as a partner. Furthermore, some participants did not make/keep records of their organizations’ dissemination of interventions thus limiting their ability to comment on dissemination effectiveness. There may also be biased introduced in the interview and data analysis since the interviewer was known to participants. However, we have taken the necessary steps to promote reflexivity and trustworthiness, such as the use of reflexive journals by the researchers and regular team discussions to critically examine personal biases and having an independent researcher (with no prior relationship to the partner organizations) involved in the data analysis process’.

## Conclusion

The dissemination and uptake of evidence-based interventions can be a slow process, and one which may be sped up by developing and subsequently leveraging key organizational partnerships. Interviews conducted produced rich data relating to partner organization’s experiences and views on potential improvements to the dissemination strategy for the *TransformUs* initiative. Researchers planning to form research-practice partnerships should consider the organizational barriers their partners may face during dissemination efforts and plan to support partner organizations through regular communication and the provision of relevant data to help form positive research–practice experiences. The dissemination strategy should be tailored to end-user audiences’ preferences and needs. The outcomes of this study will inform the further refinement of the scale up of *TransformUs* to increase adoption of the program across the state.

## Data Availability

The datasets generated and/or analysed during the current study are not publicly available due to a lack of organizational consent. They are available from the corresponding author on reasonable request and approval from the Deakin University Human Research Ethics Committee, the Victorian Department of Education and Training, the NSW Department of Education, the Australian Catholic University, and the relevant Catholic Education Offices.

## Transparency Statement

**Study Registration**: The trial was registered at the Australian New Zealand Clinical Trials Registry (ANZCTR), Number: ACTRN12617000204347. **Analytic Plan Pre-Registration**: The analytic plan was registered as part of the trial registration at the Australian New Zealand Clinical Trials Registry, Number: ACTRN12617000204347. **Analytic Code Availability**: There is no analytic code associated with this study. **Materials Availability:** Materials used to conduct this study are available as Appendices.

## Appendix 1. *TransformUs* implementation, dissemination, and scale-up strategies (Table adapted from Koorts et al. [24])

**Table:**

#	Strategy	Definition	Actors *(those who deliver imp strategy)*	Action *(specific action or process)*	Action target *(who it’s meant to affect)*
1	Formative work with key parties	Research–practice partnership to identify strategies, barriers/facilitators to program dissemination, implementation, and sustainability at scale	*TransformUs* research team with State-level partner orgs (support system)	Multiple stakeholder workshops to explore aspects of the support system and delivery context Co-develop resources and strategies for implementation and scale up	State-level partner orgs (system level) School leaders and teachers (org/implementer level)
2	Creation of coalitions and networks for program/policy advocacy	Active engagement with State education decision-makers, engaging opinion leaders (in government and non-government) to support and endorse implementation	State-level partner orgs (support system)	Consultation with key state-level partners and decision-makers to align program with state-level targets (e.g. Vic Education State target)	State-level partner organizations (system level) School leaders and teachers (delivery system)
3	Utilize multiple dissemination routes/channels	Program dissemination and promotion will occur via multiple channels known to have high reach among relevant decision-makers	State-level partner orgs (support system) School leaders and teachers (delivery system)	Program launch with media involvement. Dissemination via web links, email listserves, newsletters, teacher prof. learning networks, conferences, and workshops	*TransformUs* School leaders and teachers (delivery system)
5	Online platform for program materials and training	All program materials, training, resources, and data collection housed within an online platform aimed at schools and teachers. Schools and teachers must register to access training and materials	*TransformUs* research team with State-level partner orgs (support system)	Website hosted and maintained by *TransformUs* research team; link disseminated by all partner orgs.	School leaders and teachers (delivery system) Parents of children at *TransformUs* schools
6	Enable implementation flexibility and contextual adaptation	Non-prescriptive approach to implementation. Schools and teachers encouraged via training and in resources to adapt program strategies for setting relevance	*TransformUs* research team with State-level partner orgs (support system)	Resources include modifiable lesson plans and ‘example’ ways of delivering strategies (e.g. active breaks). Training videos illustrate ways of adapting program to different contexts	School leaders and teachers (delivery system)
7	Enable both ‘top-down’ and ‘bottom-up’ program adoption	School-level adoption not required for teacher-level implementation. Schools and teachers can register to deliver the program independently	*TransformUs* research team with State-level partner orgs (support system)	Schools and teachers register via the program website. At registration, schools encouraged to invite all teachers, and teachers encouraged to advocate for senior leadership support. Parents can advocate for school adoption. Template email invites provided	*TransformUs* School leaders, teachers (delivery system) Parents of children at *TransformUs* schools
8	Utilize existing resources in the delivery system	Program strategies can use existing school resources, equipment, and facilities where appropriate	*TransformUs* research team with State-level partner orgs (support system)	Program training and resources include ways of using/adapting existing school resources and delivering program within existing schools’ infrastructure to achieve effective implementation	*TransformUs* School leaders, teachers (delivery system)
10	Alignment with existing state-level initiatives and guidelines	Program aligned with the Victorian Achievement Program (AP) to count toward AP physical activity benchmarks for schools. Program materials (e.g. health lessons) aligned with the Victorian Curriculum	*TransformUs* research team with State-level partner orgs (support system)	*TransformUs* included as part of AP program materials and promotion. Alignment with Victorian Curriculum promoted via website and in training	*TransformUs* School leaders, teachers (delivery system)
11	Promote use of program champions	Schools identify champion(s) who advocate for *TransformUs* and are a point of contact for staff, students, and families regarding *TransformUs* implementation	*TransformUs* champion/teachers (delivery system) State-level partner orgs (support system)	Template champion position description provided to schools after registration. Online training encourages teachers to self-nominate	*TransformUs* School leaders, champion/teachers (delivery system)
14	Monitoring and evaluation to adjust scaling strategy, feedback to support schools	Multilevel data (system, org., implementer, and recipient level), on partner orgs. dissemination activities (type, freq., and dose) and setting-level implementation	*TransformUs* research team and state-level partner orgs (support system) School leaders and teachers (delivery system)	6 monthly monitoring of partner org. dissemination activities Recruitment for interviews and surveys embedded within program website	State-level partner organizations (support system) School leaders and teachers (delivery system) Parents of children at *TransformUs* schools

## Appendix 2. *TransformUs* partner organization interview questions

### Instructions: To Be Read to Participant

You have been invited to participate in this interview as you are a representative of one of the partner organizations supporting *TransformUs* roll out across Victoria. This discussion should last no more than 30 minutes. Questions will relate to your experiences promoting and disseminating *TransformUs* via your networks. Please speak freely and honestly about your experiences, both positive and negative, of being involved in the implementation of this project, and also being involved in a research partnership. Please feel free to respond on behalf of yourself and/or your organization.

This interview will be audio-recorded, transcribed, and coded anonymously for analysis. Your name and organization will not be used in any of the published material and your responses will remain unidentifiable. You will have the opportunity to read and edit the transcript of this interview. Please ensure you have read the PLS and asked any questions before we begin.

*Signed consent must have been received prior.*

#### Researcher

I want to start by asking you a few basic questions about yourself.

##### Descriptive

Which organization do you represent and what is your current job role?

How long have you held this position?

What has been your involvement with *TransformUs*?

(Prompt: e.g. development of program materials, dissemination etc.)

##### Reach

Has your organization had any involvement in promoting *TransformUs* to schools/teachers? How many schools/teachers do you expect to reach?

Has your organization had any involvement in promoting *TransformUs* to other organizations or individuals that work with schools?

Via which channels? Which information? How many teachers/schools would this have reached?

##### Effectiveness

Do you think your program dissemination strategy has been successful?

*If yes*, why?

*If no*, why not?

##### Reach

*If not obvious by organization type i.e. Independent Schools Association:*

What types of schools/teachers does your organization reach?

What proportion of the schools/teachers within your network do you anticipate will adopt *TransformUs* in the next 12 months?

Do you think *TransformUs* Is important to your organization?

*To your role?*

*If no*, why not?

*Barriers/facilitators?*

What do you think are the main challenges in your organization in promoting *TransformUs*? (*Prompt, what help to overcome this?)*

Thinking about schools now…

##### Adoption

What do you think are the major barriers and facilitators to adoption of the program in schools in general? (*Prompt if needed, what would overcome those barriers?*)

Are some schools more or less likely to adopt TransformUs over others? (Prompt if needed, what are the characteristics of these schools?)

##### Implementation

Has your organization played a role in helping schools deliver *TransformUs*?

If no, skip Q15.

What more could be done from your organization’s perspective?

Why? Why not?

Who would be responsible for organizing this?

##### Maintenance

Is your organization planning to continue supporting/endorsing/promoting (select as appropriate) the program to schools moving forward?

*If yes*, what will enable you to do this?

*If no*, why not?

*Key challenges?*

What do you think are the barriers/enablers to making *TransformUs* sustainable as a state-wide initiative?

##### Recommendations

Lastly, I would like your opinion on recommendations for improving the program moving forward.

Based on your experiences over the last year, can you recommend ways to:

*(Select as appropriate based on previous answers)*

Increase uptake of the program among schools

Make the program more likely to have positive outcomes

*(Prompt, on children/teachers/school)*

Improve implementation of the program in practice

Increase the likelihood it becomes embedded within VIC government/school system

Apart from our partners, are there any other key organizations who should be on-board?

State/national perspective

Now I would like to talk about the research partnership!

Is there anything you would like to see from us to help you promote the program within your organization or external to your organization?

How would you describe your experience of being involved in the research project?

What has worked well? Not so well?

Have you had any involvement in implementing other programs? What could we learn from this?

Have there been any other benefits from being involved? (e.g. networking)

Do you have any further comments you would like to make that we haven’t covered?

*Thank participant.*

## Appendix 3 Standards for Reporting Qualitative Research (SRQR)

**Table:**

Title and Abstract	
Title	Optimizing intervention dissemination at scale: a qualitative study of multi-sector partner organization experiences
Abstract	See ‘Abstract’ on Page 1.
Introduction	
Problem formulation	Whilst effective research-practice partnerships are essential in dissemination efforts to increase population levels of physical activity, we know less regarding how relevant parties support the dissemination of interventions, nor their potential role in increasing the likelihood of subsequent end-user adoption.
Purpose or research question	The purpose of this paper was to present findings from a qualitative evaluation of the state-wide dissemination of an efficacious primary school-based physical activity and sedentary behaviour intervention (*TransformUs*) across Victoria, Australia.
Methods	
Qualitative approach and research paradigm	A qualitative study involving semi-structured telephone interviews with key contacts representing *TransformUs* partner organizations was conducted (*n* = 15).
Researcher characteristics and reflexivity	Two independent coders kept a research journal throughout the analysis to aid reflexivity (i.e. the research process and the researchers position within this context). To promote trustworthiness of the coding and analysis process, an independent researcher (MC) who has no prior relationship to the partner organizations provided an additional layer of objectivity and helped to validate the findings. Regular debriefing sessions were conducted with the independent researcher and the primary research team to reflect on the coding process, discuss potential biases, and refine the analytical framework.
Context	See ‘Overview of *TransformUs’* on Page 4.
Sampling strategy	See ‘Participants’ on Page 5.
Ethical issues pertaining to human subjects	The *TransformUs* trial has been approved by the Deakin University Human Research Ethics Committee (HEAG-H 28_2017), the Victorian Department of Education and Training, the NSW Department of Education, Australian Catholic University (2017-145R), and the relevant Catholic Education Offices.
Data collection methods	Interviews were conducted via telephone by a trained qualitative researcher and lasted between 25 and 50 minutes (Mean = 38 minutes).
Data collection instruments and technologies	All interviews were voice-recorded and transcribed verbatim by an external online transcription company.
Units of study	See ‘Table 1. Participant characteristics’
Data processing	Interviews were audio-recorded, transcribed, and coded anonymously
Techniques to enhance trustworthiness	Coding discrepancies were resolved by discussion and consensus decision.
Results/findings	
Synthesis and interpretation	Four themes and 13 sub-themes emerged from the interview data (Fig. 1), which related to (i) organizational barriers and facilitators to dissemination; (ii) requested implementation support for partners; (iii) research–practice partnership experience; and (iv) the dissemination strategy.
Links to empirical data	Each of the four themes was reported in ‘Results’ section, with supporting quotes (Page 7–16).
Discussion	
Integration with prior work, implications, transferability, and contributions to the field	This study explored the process of ongoing dissemination of an evidence-based intervention being offered at scale, from the perspective of key individuals from partner organizations. Findings highlight alignment between the dissemination process experienced by partner organizations and the scale-up and implementation strategies
Limitations	See ‘Strengths and limitations’ on Page 20.
Other	
Conflicts of interest	The authors declare they have no competing interests.
Funding	This trial is funded by a 5-year NHMRC Partnership Grant (APP 1115708). The funding bodies had no input into the study design, data collection, or decision to publish.
